# Retraction Note: LncRNA HAND2-AS1 exerts anti-oncogenic effects on bladder cancer via restoration of RARB as a sponge of microRNA-146

**DOI:** 10.1186/s12935-025-03936-2

**Published:** 2025-08-07

**Authors:** Liping Shan, Wei Liu, Yunhong Zhan

**Affiliations:** 1 Department of Urology, Shengjing Hospital, China Medical University, 36 Sanhao Street, Heping District, Shenyang, 110004 Liaoning China; 2 Emergency Department, First Hospital of China Medical University, Shenyang, Liaoning China

**Retraction Note: Cancer Cell International (2021) 21:361**

10.1186/s12935-021-02063-y

The Editors-in-Chief have retracted this article. After publication, concerns were raised regarding highly similar western blot images between this and other articles [1–3], one of which is from the same author group [1]. Specifically:

- Fig. 4F RARB and GAPDH lanes 2–4 appear highly similar to Fig. 5E Mcl-1 and GAPDH in [1];
- Fig. 5D 5637 Caspase 3 blot appears highly similar to Fig. 4F Akt(p-Ser308) in [2] and Fig. 4E beta-actin in [3].

The authors have been unable to provide the full raw data for validation upon request. The Editors-in-Chief therefore no longer have confidence in the presented data.

Yunhong Zhan agrees with this retraction. Liping Shan and Wei Liu have not responded to any correspondence from the editor or publisher about this retraction.
